# Endometrial glucose metabolism during early pregnancy

**DOI:** 10.1530/RAF-23-0016

**Published:** 2023-12-13

**Authors:** Ziting Chen, Matthew Dean

**Affiliations:** 1 Department of Animal Science, University of Illinois, Urbana-Champaign, Urbana, Illinois, USA; 2 Department of Pharmaceutical Sciences, University of Michigan, Ann Arbor, Michigan, USA

**Keywords:** uterine epithelium, decidualization, glycogen, lactate, fructose, glycosylation

## Abstract

Approximately 50% of pregnancies in humans fail, mostly before or during implantation. One factor contributing to pregnancy loss is abnormal glucose metabolism in the endometrium. Glucose contributes to preimplantation embryo development, uterine receptivity, and attachment of the embryo. Across multiple species, the epithelium stores glucose as the macromolecule glycogen at estrus. This reserve is mobilized during the preimplantation period. Glucose from circulation or glycogenolysis can be secreted into the uterine lumen for use by the embryo or metabolized via glycolysis, producing ATP for the cell. The resulting pyruvate could be converted to lactate, another important nutrient for the embryo. Fructose is an important nutrient for early embryos, and the epithelium and placenta can convert glucose to fructose via the polyol pathway. The epithelium also uses glucose to glycosylate proteins, which regulates embryo attachment. In some species, decidualization of the stroma is critical to successful implantation. Formation of the decidua requires increased glucose metabolism via the pentose phosphate pathway and glycolysis. After decidualization, the cells switch to aerobic glycolysis to produce ATP. Paradoxically, the decidua also stores large amounts of glucose as glycogen. Too little glucose or an inability to take up glucose impairs embryo development and decidualization. Conversely, too much glucose inhibits these same processes. This likely contributes to the reduced pregnancy rates associated with conditions like obesity and diabetes. Collectively, a precise control of glucose metabolism is important for several endometrial processes required to establish a successful pregnancy. The factors regulating these metabolic processes remain poorly understood.

**Lay summary:**

Pregnancy failure soon after an egg has been fertilized is common in humans and cattle. The inner lining of the womb (endometrium) plays a role in the development and implantation of an embryo. The levels of glucose needed by the endometrium and embryo change dramatically during early pregnancy. The inner layer of tissue (epithelium) uses glucose and other nutrients to help the embryo develop and attach to the endometrium. In some species, the layer underneath the epithelium (stroma) goes through a series of major changes that alter the function of the cells and the levels of energy they require. This review discusses the way glucose is used in the epithelium and stroma to provide insights into the role this has in ongoing pregnancy.

## Frequency of pregnancy failure

Infertility is a common problem in reproductive health. Maximal fertility in women trying to conceive is only 30% per cycle (Zinaman *et al.* 1996, Bonde *et al.* 1998). Wilcox *et al.* (1988) found that 22% of early pregnancy losses detected utilizing a highly sensitive human chorionic gonadotropin (hCG) assay were not recognized clinically. A similar study found that 41% of pregnancies detected via urinary hCG never reached clinical diagnosis (Zinaman *et al.* 1996). Losses before hCG is secreted by the trophoblasts cannot be detected. Therefore, it is impossible to determine the total rate of pregnancy loss in humans. Nonetheless, it is estimated that ~50% of pregnancies in humans fail (Macklon *et al.* 2002, Annual Capri Workshop Group 2020).

Cattle are important agricultural species and serve as a biomedical model in certain situations (Spencer *et al.* 2022). Cattle have a relatively long preimplantation period and gestate a single fetus, like humans. This makes them a good model for understanding how the uterus supports preimplantation embryo development. Estimates of pregnancy loss in cattle agree with estimates from humans (Diskin *et al.* 2015, Reese *et al.* 2020). In dairy cows, rates of embryonic and fetal loss (excluding fertilization failure) were calculated to be between 40% and 56% (Diskin & Morris 2008). A recent meta-analysis compiled 56,000 diagnostic records spanning fertilization, early embryo, and late embryo/early fetal periods in beef cows. They found that the predicted pregnancy rate was only 50% by day 30 of gestation (Reese *et al.* 2020). Thus, in humans and cows, most pregnancies fail very early, before a functional placenta has developed.

To successfully implant, complex interactions between a viable embryo and an appropriately primed uterus are necessary (Dey *et al.* 2004, Ng *et al.* 2020). Dysregulation in many processes or signaling pathways in embryonic development or uterine receptivity plays a part in low pregnancy rates (Dey *et al.* 2004). One factor linked to pregnancy loss is abnormal uterine metabolism (Maeyama *et al.* 1977, Wiebold 1988, Wang *et al.* 2021, Chen *et al.* 2023, Yang *et al.* 2023*a*). The nutritional needs of the embryo change dramatically prior to and during implantation (Hardy *et al.* 1989, Gardner *et al.* 1993). To meet the changing needs of the embryo, the uterine epithelium takes nutrients from maternal circulation, potentially store or metabolize them, and then secretes the needed nutrients into the uterine lumen. In addition, processes such as cellular proliferation, decidualization, and embryo attachment dramatically change the nutritional needs of the endometrium. The most studied nutrient for both embryo development and endometrial metabolism is glucose. This review aims to summarize what is known about glucose metabolism in the endometrium and highlight potential ways altered glucose metabolism could contribute to reduced fertility.

## Maternal metabolism and fertility

It is clear that changes in maternal metabolism affect reproduction. Two of the best examples in humans are diabetes and obesity; both are associated with lower fertility (Temple *et al.* 2002, Bellver 2022). Diabetes and obesity can affect the reproductive system in many different ways. For example, zygotes transferred from diabetic to healthy mice show fewer implantation sites and lower fetal weights than zygotes from healthy controls, indicating that diabetes affects the oocyte or zygote directly (Wyman *et al.* 2008). There is also substantial evidence that obesity directly affects the uterus. Obesity is associated with lower fertility in women receiving donated ova or serving as surrogates (DeUgarte *et al.* 2010, Bellver *et al.* 2013). Type 2 diabetes and obesity are associated with hyperinsulinemia, hyperglycemia, and other systemic changes in metabolism, any of which could contribute to uterine dysfunction (Lathi *et al.* 2005, Khaliq *et al.* 2020, Berg *et al.* 2022). In mice, diet-induced obesity dysregulates glycolysis in the endometrium, impairs decidualization, and reduces litter size (Chen *et al.* 2023).

Another example of maternal metabolism affecting fertility is lactation. High levels of milk production in modern dairy cows reduce fertility (Lucy 2001, Maillo *et al.* 2012). And embryo transfer experiments have shown that lactation compromises the ability of the uterus to support early embryo development independently of any effects on the oocyte (Maillo *et al.* 2012). Interestingly, lactation has effects generally opposite of type 2 diabetes and obesity on maternal metabolism. The onset of lactation is associated with decreases in serum glucose and insulin concentrations (Forde *et al.* 2015). Surprisingly, little is known about how changes in the concentrations of insulin, glucose, and other regulators of metabolism affect carbohydrate metabolism in the uterus.

## Overview of glucose metabolism

**Figure 1:**
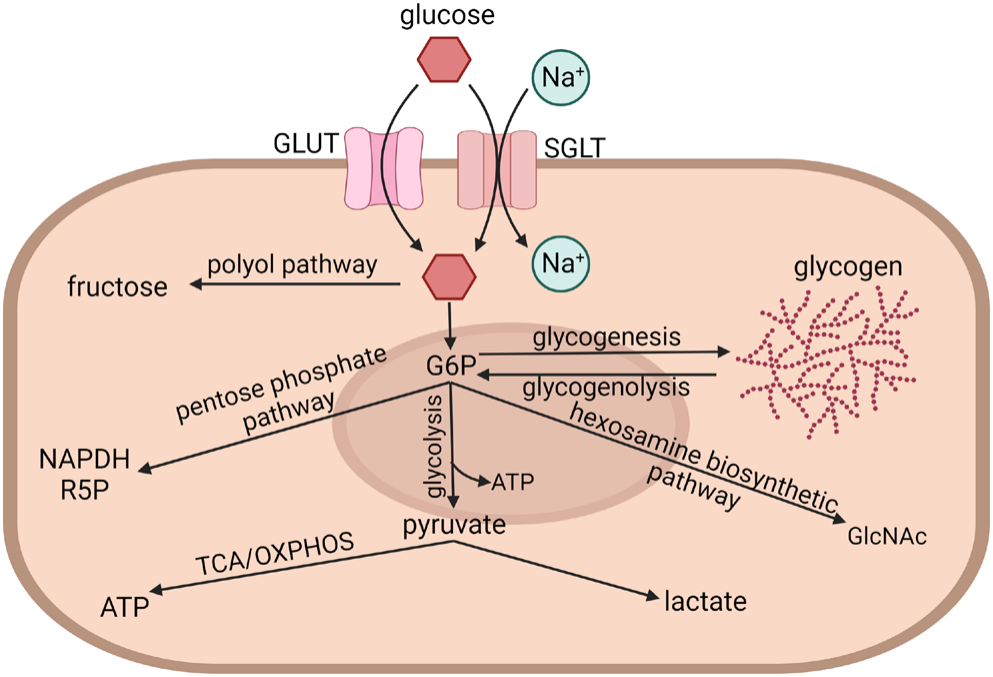
Overview of significant glucose metabolic pathways. Glucose enters a cell through facilitative glucose transporters (GLUTs) or sodium–glucose linked transporters (SGLTs). Intracellular glucose can be converted to fructose by the two-step polyol pathway or phosphorylated by hexokinase, forming glucose-6-phosphate (G6P). G6P can transiently be stored as glycogen via glycogenesis. G6P is liberated from glycogen via glycogenolysis. G6P can enter glycolysis, producing two molecules of ATP and two molecules of pyruvate. Pyruvate can be converted to lactate or fed into the tricarboxylic acid (TCA) cycle and oxidative phosphorylation (OXPHOS), producing ATP. G6P can also enter the pentose phosphate pathway, producing NADPH and ribose-5-phosphate (R5P). G6P can also enter the hexosamine biosynthetic pathway, producing *N*-acetylglucosamine (GlcNAc), which is used to glycosylate proteins. Created with BioRender.com.

To enter or exit a cell, glucose must pass through a glucose transporter. GLUTs (gene family *SLC2A*) transport glucose via facilitative diffusion. It should be noted that some GLUTs transport other molecules in addition to glucose, and some do not transport glucose at all (Ismail & Tanasova 2022). Glucose can also enter a cell through sodium–glucose linked transporters (SGLTs; gene family *SLC5A*). SGLTs are secondary active transporters that use sodium to bring glucose into cells (Fig. 1).

Once inside a cell, glucose can be converted to fructose via the polyol pathway or phosphorylated by hexokinase, producing glucose-6-phosphate (G6P). G6P can be metabolized by four major pathways: glycolysis, which produces ATP and pyruvate; the pentose phosphate pathway (PPP), which produces NADPH and pentoses (five-carbon sugars) usually to support cell proliferation; the hexosamine biosynthetic pathway (HBP), which produces substrates for protein glycosylation; and glycogenesis, which temporarily stores glucose as part of glycogen (Fig. 1).

## Nutritional needs of preimplantation embryos

Fertilization happens in the ampulla of the fallopian tubes. Cleavage development then proceeds as the embryo moves toward the uterus. In humans, the embryo enters the uterus as a morula 4–5 days after fertilization, and implantation occurs 7–10 days post fertilization (Bigelow 1977, Wilcox *et al.* 1999). Prior to implantation, embryos are dependent on the secretion of growth factors and nutrients, collectively known as histotroph. Evidencing the importance of histotrophic secretions, preventing uterine gland formation leads to embryo death in sheep and mice (Gray *et al.* 2001, Dunlap *et al.* 2011, Filant & Spencer 2013).

Cattle form synepitheliochorial placentas, meaning that giant binucleate cells within the chorion fuse with the uterine surface epithelium. However, the placenta never invades through the basal lamina, indicating that histotrophic nutrition is important throughout gestation (Green *et al.* 2021). In contrast, humans have an invasive hemochorial placenta, and it takes time to establish contact with maternal circulation. Histological examinations, oxygen tension measurements, and ultrasonography data suggested that blood flow to the intervillous space is not fully established until the end of the first trimester (Jaffe & Woods 1993, Burton *et al.* 1999, 2002, Jauniaux *et al.* 2001). An RNA-seq study comparing first- and second-trimester human placenta found a higher expression of hexokinase 2 (*HK2*) and pyruvate kinase L/R (*PKLR*) during the first trimester, suggesting that the placenta is more reliant on glycolysis during this period (Prater *et al.* 2021). Thus, secretions by the uterine epithelium are likely important throughout the first several weeks of human pregnancies.

Cleavage-stage embryos primarily use lactate and pyruvate for energy. From the zygote to the 16-cell stage, pyruvate uptake was approximately 10-fold higher than glucose uptake in sheep embryos. At the morula-to-blastocyst transition, glucose uptake increased 50-fold while pyruvate uptake increased about 3-fold so that the hatched blastocyst was taking up equal amounts of pyruvate and glucose (Gardner *et al.* 1993). In cows, oxidation of radio-labeled lactate, pyruvate, and glucose increased during the transition of morulas to blastocysts, though oxidation of both lactate and pyruvate remained somewhat lower than that of glucose (Khurana & Niemann 2000).

In human embryos, glucose uptake continually increased from days 2 to 6 in embryos that reached the blastocyst stage. However, glucose uptake started to increase but then decreased in embryos that were arrested in development. Glucose uptake decreased steadily in unfertilized oocytes (Hardy *et al.* 1989). Similarly, higher glucose uptake on days 4 and 5 during *in vitro* fertilization (IVF) was associated with a higher rate of establishing a pregnancy post transfer (Gardner *et al.* 2011).

Single-cell sequencing of human embryos found dramatic increases in glycolytic enzymes such as hexokinase 2 (*HK2*), phosphofructokinase liver (*PFKL*), phosphofructokinase muscle (*PFKM*), pyruvate kinase M1/2 (*PKM*), and lactate dehydrogenase A/B (*LDHA* and *LDHB*) at the eight-cell or morula stage compared to two- and four-cell embryos (Zhao *et al.* 2018). In agreement, a single-cell transcriptomic analysis combining data from six mammalian species found evidence for a switch from oxidative phosphorylation toward glycolytic metabolism during early gastrulation (Malkowska *et al.* 2022).

Collectively, these studies show that glucose consumption increases sharply as the embryo enters the uterus. However, elevated glucose metabolism may not universally be a sign of embryo competence. It has been suggested that defects in the cells of the embryo may result in increased metabolism of glucose, lipids, or amino acids and result in lesser viability (Leese *et al.* 2007). If there is an ideal level of metabolism, it likely varies by species, development stage, and concentrations of available nutrients.

There is accumulating evidence that fructose may be an important nutrient for preimplantation embryos. Studies measuring the concentration of carbohydrates in fetal and placental fluids have found that fructose concentrations are consistently higher than glucose concentrations in ungulate species (Goodwin 1956, Zavy *et al.* 1982, Bertolini *et al.* 2004). One study has extended this observation to include humans (Jauniaux *et al.* 2005). Functionally, two studies culturing bovine embryos in equal concentrations of glucose or fructose found higher rates of blastocyst development with fructose (Kimura *et al.* 2005, Barceló-Fimbres & Seidel 2007). It was also found 5.6 mM of fructose did not skew the ratio of surviving embryos toward males like an equal concentration of glucose did (Kimura *et al.* 2005). Day 16 pig conceptuses could metabolize glucose or fructose when only one substrate was available. However, when cultured with both sugars, they preferentially oxidized glucose (Kramer *et al.* 2020). Hence, either glucose or fructose can support embryo development, but the role(s) of each monosaccharide remain to be fully elucidated.

## Metabolism and secretion of nutrients by the uterine epithelium

### Secretion of glucose into the uterine lumen

Of the 14 known GLUTs, 9 have been detected in the uterus (Frolova & Moley 2011). Factors regulating the localization and expression of various GLUTs are an active area of investigation. For a detailed review of the role of GLUTs in uterine function, we recommend several reviews on the topic (Frolova & Moley 2011, Vrhovac Madunić *et al.* 2021).

Given near ubiquitous expression of GLUTs in the uterine epithelium and their mechanism via facilitated diffusion, it might be expected that glucose freely diffuses through the epithelium and into the uterine lumen (Frolova & Moley 2011). However, several pieces of evidence indicated that the amount of glucose entering the lumen is tightly regulated. In humans, mice, and cows, glucose concentrations are consistently lower in the uterine fluid than in systemic circulation (Wales & Edirisinghe 1989, Gardner *et al.* 1996, Hugentobler *et al.* 2008). The concentration of glucose in the uterine fluid has been determined in a number of species under different conditions. In humans, Gardner *et al.* (1996) found that glucose concentrations in the uterine fluid averaged 3.15 mM and did not vary across the cycle. In mice, glucose concentrations at estrus were found to be only 0.6 mM (Harris *et al.* 2005). In cattle, glucose concentrations were determined in uterine fluid on day 6, 8, and 14 of the cycle (Hugentobler *et al.* 2008); glucose concentrations did not change and averaged 4 mM. Plasma glucose concentrations were 6.5 mM. This agrees with *in vitro* studies using bovine embryos showing that serum concentrations of glucose impairs bovine embryo development (Kimura *et al.* 2005). In heifers, plasma glucose and uterine fluid glucose concentrations are not correlated (Moraes *et al.* 2019). Even intravenous infusion of glucose into Holstein dairy cows failed to alter glucose concentrations in uterine fluid (Leane *et al.* 2018). Weibold (1988) collected embryos and uterine fluid from cows on day 7 of pregnancy. Cows with morphologically abnormal embryos also had 2-fold higher glucose concentrations in their uterine fluid. These results suggest that glucose secretion into the uterine lumen is tightly regulated.

### Production of lactate and ATP

**Figure 2:**
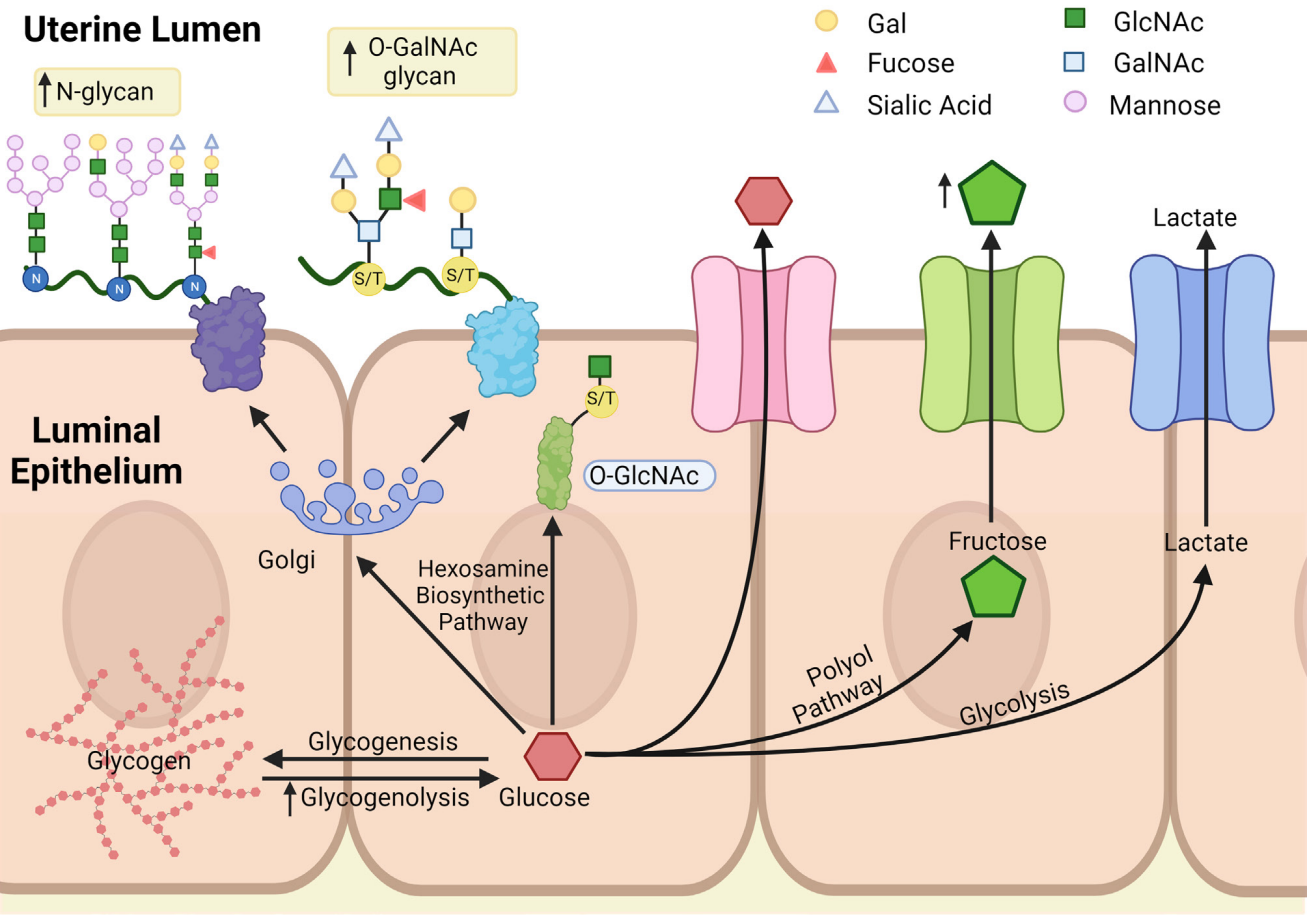
Summary of glucose metabolism in uterine epithelium. Once glucose is inside the epithelium, it can be stored as glycogen through glycogenesis and liberated from glycogen by glycogenolysis. Glucose can also be converted to fructose and lactate through polyol pathway or glycolysis, respectively, which can be transported into the uterine lumen. The hexosamine biosynthetic pathway converts glucose into *N*-acetylgalactosamine, which is the substrate for glycosylation. Arrows represent changes associated with pregnancy. Gal, galactose; GalNAc, *N*-acetylgalactosamine; GlcNAc, *N*-acetylglucosamine; N, asparagine; S/T, serine/threonine. Created with BioRender.com.

The uterine epithelium could prevent glucose from freely diffusing into the uterine lumen and provide other nutrients used by the embryo through the metabolism of maternal glucose (Fig. 2). Glycolysis catabolizes glucose into two pyruvate molecules while generating a net of two ATP molecules. Pyruvate can easily be converted to lactate by lactate dehydrogenase. In humans and cattle, lactate concentrations in the uterine fluid are higher than in serum and do not change during the cycle, indicating that lactate is actively exported by the uterine epithelium (Gardner *et al.* 1996, Hugentobler *et al.* 2008). In agreement, progesterone supplementation did not affect lactate or pyruvate concentrations in the uterine lumen of cattle (Hugentobler *et al.* 2010). Very little seems to be known about the expression of lactate transporters (primarily MCT1 (*SLC16A4*) and MCT4 (*SLC16A4*)) in the endometrium (Zuo *et al.* 2015).

Pyruvate can also enter the TCA cycle, generating 32 more ATP molecules. Little is known about factors controlling flux through glycolysis in the uterine epithelium. The uterine epithelium expresses high levels of hexokinase 1, which phosphorylates glucose, yielding G6P (Sandoval *et al.* 2021, Chen *et al.* 2022). However, G6P is a substrate for all the major pathways that use glucose. Chase *et al.* (1992) confirmed that endometrial biopsies from cattle could metabolize glucose into lactate or oxidize it into CO_2_. In the mid-luteal phase (day 11), metabolism to lactate was lowest, and oxidation was highest. Though, that study did not include tissues collected near estrus, making it difficult to infer changes due to ovarian steroids. In addition, estradiol treatment increased glucose utilization and metabolism via glycolysis in uterine homogenates in immature rats (Baquer & McLean 1972). Another study found that estradiol stimulated IGF1 in the uterine stroma of cows, which agrees with studies from mice (Cooke *et al.* 1997, Gonzalez *et al.* 2022). IGF1 increased the levels of 3-phosphoglycerate and lactic acid in immortalized bovine uterine epithelial (BUTE) cells (Gonzalez *et al.* 2022). Collectively, these results suggest that estradiol increases glucose uptake and glycolytic metabolism by the uterine epithelium, perhaps indirectly.

### Conversion of glucose to fructose in the uterine epithelium

Fructose is readily detected in fetal and placenta fluids from various species, including cattle and humans (Goodwin 1956, Zavy *et al.* 1982, Bertolini *et al.* 2004, Jauniaux *et al.* 2005). Given that fructose is undetectable in maternal serum, fructose likely originates from glucose in the maternal circulation. Conversion of glucose to fructose could also contribute to limiting how much glucose is present in the uterine lumen.

Glucose can be converted into fructose in the two-step polyol pathway. First, glucose is converted to sorbitol via aldose reductase (AKR1B1). Sorbitol dehydrogenase (SORD) then converts sorbitol into fructose. In pigs and mares, the amount of recovered fructose is much higher in the pregnant uterus than in the cyclic uterus (Zavy *et al.* 1982). In the pig uterus, AKR1B1 and SORD are detectable in the uterine epithelium by immunohistochemistry as early as day 5 and day 13 of pregnancy, respectively, showing that the uterine epithelium can convert maternal glucose into fructose. Interestingly, endometrial AKR1B1 expression peaked on day 12 in pregnant gilts and was 50-fold greater than day 12 of the estrous cycle, suggesting that pregnancy increases the production of fructose by the uterine epithelium. On day 12 of pregnancy, porcine embryo elongates morphologically and produces estrogen that functions as the signal of maternal recognition. Explant culture showed that estrogen (the maternal recognition factor in pigs) increased *AKR1B1* mRNA levels, and expression of *AKR1B1* was higher in explants with elongated embryos than spherical embryos. This suggests that the increase of AKR1B1 is likely caused by estrogen produced by the embryo (Seo *et al.* 2014). The concentration of both carbohydrates increased significantly in the uterine flush after day 12 of pregnancy (Zavy *et al.* 1982). After the attachment of the embryo, the trophectoderm expresses much higher levels of AKR1B1 and SORD than the uterus. Thus, the placenta probably takes over fructose synthesis (Steinhauser *et al.* 2016). These results indicate that prior to implantation, the uterine epithelium converts glucose into fructose; however, the precise roles of fructose remain unclear.

### Protein glycosylation and glycation in the uterine epithelium

Another vital role of glucose is the glycosylation of proteins. Glycosylation is a post-translational modification where carbohydrate molecules are attached to proteins and regulate protein function. Broadly, there are two major types of glycosylation. In the first type, carbohydrates are added to proteins during synthesis. These include O-N-acetylgalactosamine (O-GalNAc) glycosylation and *N*-glycosylation. These glycans are only added to extracellular and transmembrane proteins. Once added to a protein, *N*- and *O*-GalNAc glycans remain relatively unchanged during the life of the protein (Brockhausen *et al.* 2009).

The apical surface of the uterine epithelium and trophoblasts are heavily glycosylated, and this glycosylation is important in embryonic attachment (Zhu *et al.* 1995, Clark 2015). In rabbits, glycoprotein expression on the luminal epithelium was higher in pseudopregnant animals compared to animals at estrous (Anderson *et al.* 1986). In humans, glycosylation on the surface of the uterine epithelium increased during the window of implantation (Genbacev *et al.* 2003, Yu *et al.* 2019). Functionally, inhibition of Lewis Y (Ley) glycans with antibodies prevented implantation (Zhu *et al.* 1995), and exogenous lacto-*N*-fucopentaose I, an oligosaccharide, inhibited embryo attachment in mice (Lindenberg *et al.* 1988). Modeling attachment of the embryo *in vitro* and reducing *N*-linked glycosylation decreased binding of syncytiotrophoblast-like JAR cells to both Ishikawa and RL95-2 cells. Similarly, the reduction of *N*-linked glycosylation in the uterine lumen reduced the number of implantation sites in mice (Yu *et al.* 2019). Collectively, these results highlight the importance of *N*-glycans and O-GalNAc glycans in the attachment of the embryo to the uterus (Fig. 2).

Few studies have identified specific proteins that are glycosylated on the surface of the uterine epithelium. Yu *et al.* (2019) found that treating Ishikawa cells with tunicamycin (inhibits GlcNAc phosphotransferase) reduced *N*-glycosylation of integrin αvβ3 and leukemia inhibitory factor receptor (LIFR) by altering protein migration in acrylamide gels. Both tunicamycin and peptide-*N*-glycosidase F (PNGase F, removes *N*-glycans) reduced FAK/paxillin signaling and diminished the ability of JAR cells to attach to Ishikawa cells. Tunicamycin and PNGase also inhibited the phosphorylation of STAT3 following LIF treatment. More glycosylated proteins on the surface of the uterine epithelium remain to be identified.

Species differences in glycan expressions on the uterine epithelium may contribute to the species specificity in the ability of the embryo to attach to the uterine epithelium, a hypothesis known as the glycocode (Jones & Aplin 2009). The surfaces of the uterine epithelium and trophoblasts are known to be heavily glycosylated (Jones *et al.* 1997). The glycans present at the maternal–fetal interface differ across species. However, species that can interbreed also have more similar glycans present (Jones *et al.* 2000). If the glycocode hypothesis is correct, that would suggest that glucose metabolism (i.e. the specific glycans produced by the uterine epithelium) determines the species specificity of embryonic attachment and implantation.

The second major type of glycosylation is *O*-linked *N*-acetylglucosamine glycosylation (*O*-GlcNAcylation). In this type of glycosylation, nuclear or cytosolic proteins are glycosylated due to the actions of *O*-GlcNAc transferase (OGT) or endoplasmic reticulum (ER)-resident enzyme *O*-GlcNAc transferase (EOGT). OGT glycosylates more than 3000 proteins, while EOGT is thought to only target a few hundred (Groves *et al.* 2013, Varshney & Stanley 2017). Proteins are deglycosylated by *O*-GlcNAcase (*OGA*). Proteins can be repeatedly glycosylated and deglycosylated, suggesting that *O*-GlcNAc may dynamically regulate protein function (Fig. 2). Supporting that view, many of the serine or threonine residues that are glycosylated are also phosphorylated, suggesting that these two posttranscriptional modifications compete to jointly regulate protein activity (Brockhausen *et al.* 2009).

In the uterine epithelium, levels of *O*-GlcNAc-modified proteins were higher during the secretory phase in human samples than in the proliferative phase. RNA interference of OGA demonstrated higher cell proliferation, migration, and invasion abilities, whereas knockdown of OGT resulted in decreased migration (Han *et al.* 2019). *In vitro,* the knockdown of OGT also decreased the proliferation and invasion of RL95-2 cells. Intraluminal injection of *OGT* siRNA resulted in fewer implantation sites in mice (Zhang *et al.* 2022). These results showed that *O*-GlcNAcylation played an important role in promoting embryo implantation. A recent study found that IGF1 treatment of BUTE cells resulted in increased levels of *N*-acetyl-glucosamine (substrate for *O*-GlcNAcylation) and increased protein glycosylation as determined by Periodic acid–Schiff (PAS) staining (Gonzalez *et al.* 2022). In mice, inhibition of OGA by thiamet G promoted trophoblast differentiation and increased breaching of the endometrial epithelium in an *in vitro* model (Ruane *et al.* 2020). EOGT expression increased during decidualization of primary endometrial stromal cells (EnSC), and knockdown of EOGT disrupted the expression of numerous genes related to energy homeostasis (Muter *et al.* 2018). Collectively, glycosylation mediates cell function in both the epithelium and trophoblasts, regulating embryonic implantation.

### The uterine epithelium and glycogen storage

The uterine epithelium can also store glucose as glycogen (Fig. 2). Glycogen is a macromolecule composed of glucose moieties linked together via α(1→4) and α(1→6) bonds. Each glycogen molecule can store up to 50,000 glucose residues; however, most molecules have a fraction of that. That way, glucose can be quickly added to or released from glycogen to match the needs of the cell. Glycogen is mostly studied in the liver and the skeletal muscle, but it is clear that the uterus can also store glycogen (Dean 2019).

Glycogen has been observed in the luminal and glandular epithelium of cows, mink, humans, and mice via histological staining and transmission electron microscopy (Demir *et al.* 2002, Dean *et al.* 2014, Jones *et al.* 2015, Sandoval *et al.* 2021, Chen *et al.* 2022). Confirming this, the glycogen-synthesizing enzyme, i.e. glycogen synthase, is highly expressed in the uterine epithelium of humans, mink, mice, and cows (Dean *et al.* 2014, Jones *et al.* 2015, Sandoval *et al.* 2021, Chen *et al.* 2022).

In mice and mink, glycogen content of luminal and glandular epithelium was high during proestrus/estrus than during pregnancy (Dean *et al.* 2014, Chen *et al.* 2022). In cows, the glycogen content of the uterine epithelium was higher near estrus than during the luteal phase (Sandoval *et al.* 2021). In agreement, estradiol stimulates the production of IGF1 in the uterine stroma, and a study showed that IGF1 increases glycogen levels in BUTE cells (Cooke *et al.* 1997, Gonzalez *et al.* 2022). Insulin has also been shown to increase glycogen levels in uterine epithelial cells (Dean and Rose 2018, Berg *et al.* 2022). This might indicate that hyperinsulinemia increases uterine epithelial glycogen storage. However, limited evidence indicates that obesity results in insulin resistance in the uterine epithelium (Mioni *et al.* 2004, Fornes *et al.* 2010). The actual effects of diabetes and obesity on glycogen metabolism in the uterine epithelium have yet to be determined.

Glycogen phosphorylase is expressed in the uterine epithelium of multiple species (Jones *et al.* 2015, Sandoval *et al.* 2021, Chen *et al.* 2022). Hence, G6P liberated from glycogen could be used in the epithelial cells. Little research has explored the hormones stimulating glycogenolysis in the uterine epithelium. Decreasing glycogen levels during pregnancy in mink and mice or during the luteal phase in cattle points toward a role for progesterone (Dean *et al.* 2014, Sandoval *et al.* 2021, Chen *et al.* 2022). In agreement, progesterone reduces glycogen levels in the uterine epithelium of mink (Bowman & Rose 2016, Hodonu *et al.* 2019). The epithelium also expresses glucose-6-phosphatase, which would dephosphorylate G6P. The glucose resulting from this reaction could exit the epithelium through GLUTs and support embryo development (Sandoval *et al.* 2021, Chen *et al.* 2022).

Another possibility is that the epithelium releases intact glycogen into the uterine lumen. A study in humans observed glycogen in the glandular lumen and intravillous space during pregnancy using both histology and electron microscopy (Burton *et al.* 2002, 2011). In agreement, diastase-labile PAS staining, indicative of glycogen, has been observed in the glandular lumen of mink and cows (Dean *et al.* 2014, Sandoval *et al.* 2021). However, PAS staining could be due to the sloughing of epithelium during processing and fixation, and one study on the human uterus failed to observe glycogen in the lumen of decidual glands (Jones *et al.* 2015).

Electron microscopy has shown that glycogen accumulates on the apical side of the epithelium during pregnancy (Demir *et al.* 2002). Two electron microscopy studies observed what appeared to be microvesicles that contained glycogen budding off from the uterine epithelium (Cornillie *et al.* 1985, Demir *et al.* 2002), indicating that extracellular vesicles released into the uterine lumen could contain glycogen.

If released into the uterine lumen, glycogen could be catabolized into glucose in uterine fluid (Simintiras *et al.* 2022). Glycogen phosphorylase, which catalyzes the rate-limiting step in glycogen breakdown, is present in the uterine fluid. An early study detected glycogen phosphorylase activity in uterine rinsings from sheep (O’Shea & Murdoch 1970). Two studies have subjected extracellular vesicles from the uterine lumen of livestock to proteomics and identified glycogen-metabolizing enzymes (Kusama *et al.* 2018, Hong *et al.* 2023). In a retrospective study, uterine fluid was collected from women undergoing IVF. The fluid was analyzed by proteomics, and the fluid from the women who did get pregnant was compared to women who did not. The protein most strongly associated with pregnancy was glycogen phosphorylase, not only suggesting that glycogen might be catabolized in the uterine fluid but also suggesting that its abundance is linked to successful pregnancy (Azkargorta *et al.* 2018).

Glycogen could potentially be taken up intact by the embryo or placenta (Li *et al.* 2016). For example, human villous trophoblasts can take up dextran (Duval *et al.* 2018). If the embryo or placenta takes up intact glycogen, it needs to be catabolized. Human placentas (6-14 weeks of gestation) showed low and inconsistent immunostaining for glycogen phosphorylase (Jones *et al.* 2015). Glycogen can also be catabolized by α-amylase; however, results from human placentas are inconsistent. One study found some immunostaining in luminal secretions but not in syncytiotrophoblast samples (Jones *et al.* 2015). Another study found high α-amylase activity in first-trimester human placentas (Fisher & Laine 1983). Finally, glycogen could be broken down in the autophagosome by the enzyme acid α-glucosidase (Koutsifeli *et al.* 2022). Acid α-glucosidase has been detected in first-trimester chorionic villi (Grubisic *et al.* 1986). Whether these enzymes degrade glycogen synthesized in the placenta or taken up from uterine fluid is currently unclear.

## Glucose metabolism by uterine stroma

**Figure 3:**
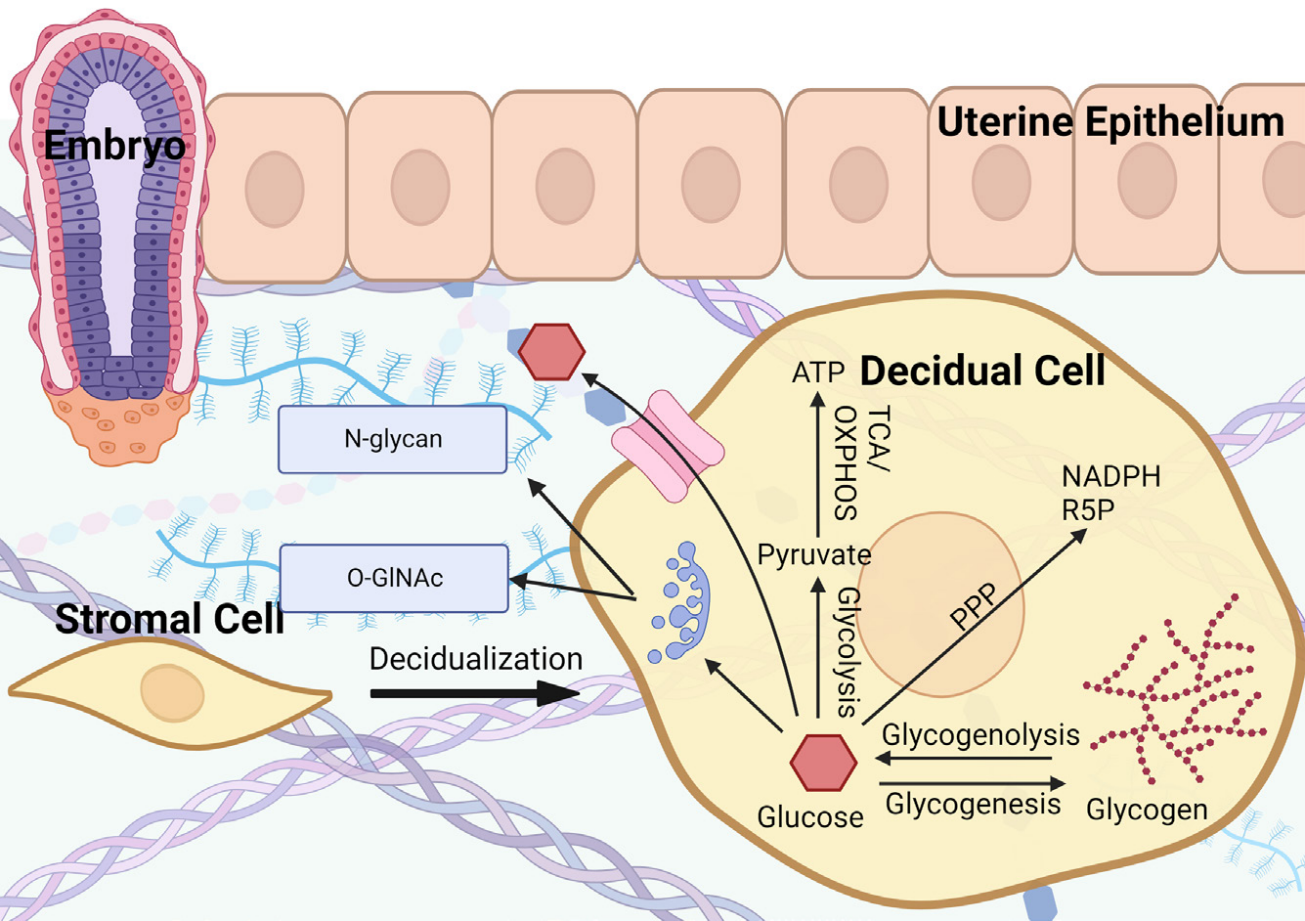
Decidualization changes glucose metabolism in the uterine stroma. During decidualization, glucose metabolism via the pentose phosphate pathway (PPP) increases to produce precursors for nucleic acids (ribose-5-phosphate (R5P)) and NADPH. After decidualization, the cell rely heavily on aerobic glycolysis, with less flux through the TCA cycle and oxidative phosphorylation (TCA/OXPHOS). During decidualization, extracellular proteins are remodeled and glycosylated (*N*-glycans and *O*-GlaNAc glycosylation). The decidua stores large amounts of glycogen. Created with BioRender.com.

In humans, the endometrium undergoes a differentiation process called decidualization (Fig. 3). The decidua, a temporary but critical structure in the uterus, is composed of differentiated endometrial stromal cells, maternal vascular cells, and a large number of maternal immune cells (Mori *et al.* 2016). Improper decidualization is clearly associated with human infertility and pregnancy complications (Salker *et al.* 2010, Garrido-Gomez *et al.* 2017, Huang *et al.* 2017). As decidualization does not occur in livestock species, research on decidualization typically uses human cells or rodent models.

### Glucose uptake in the uterine stroma

Adequate glucose is necessary for decidualization. *In vitro*, glucose concentrations less than 2.5 mM impair the decidualization of murine and human ESCs. Both GLUTs and SGLTs are found in the uterine stroma and decidua. After cells take up glucose, it is phosphorylated by hexokinase. Downregulation of hexokinase 2 (*HK2*) suppressed glucose uptake, and silencing HK2 impaired the decidual response in human endometrial stromal cells (hESCs) (Lv *et al.* 2018). Administration of 2-deoxy-d-glucose (2-DG), an inhibitor of hexokinase, inhibited the decidualization of hESCs (Kommagani *et al.* 2013).

### Decidualization and the pentose phosphate pathway

In rats, the activity of glucose-6-phosphate dehydrogenase (G6PDH; the first enzyme unique to the PPP) increased at the implantation site relative to the inter-implantation site (Moulton 1974). Functionally, inhibition of the PPP with dehydroepiandrosterone (DHEA) or glucosamine impaired the decidual response in hESCs and reduced litter size in mice (Frolova *et al.* 2011, Tsai *et al.* 2013). However, it should be noted that glucosamine also affects other aspects of fertility, such as inhibiting oocyte development through HBP upregulation (Schelbach *et al.* 2010). Huang *et al.* (2019) found that decidua from spontaneous abortions had lower levels of GPR120 (a receptor for ω-3 polyunsaturated fatty acids), and activation of GPR120 increased GLUT1 and G6PDH expression. Knockdown of *G6PDH* impaired decidualization, highlighting the importance of the PPP. Given that the PPP produces substrates that are important for cell proliferation (NADPH and five-carbon sugars), it is likely that the PPP supports the proliferation of uterine fibroblasts associated with decidualization (Fig. 3).

### Decidual glycolysis

The increase of glucose flux during decidualization may also be explained by the adaption of decidual cells to aerobic glycolysis (i.e. Warburg metabolism). Pyruvate kinase M2 (PKM2) is a glycolytic enzyme. M2 is a specific isoform of pyruvate kinase associated with Warburg metabolism. PKM2 is strongly expressed in the decidua after implantation in mice. Expression (mRNA and protein) of PKM2 increased significantly during the decidualization process both *in vivo* and *in vitro* in mice (Su *et al.* 2020). Inhibition of 6-phosphofructo-2-kinase/fructose-2, 6-bisphosphatase 3 (PFKFB3), a rate-limiting enzyme in glycolysis, suppressed decidualization in hESCs *in vitro* and attenuated decidualization in mice (Kommagani *et al.* 2013).

### Glycosylation and glycation in the decidua

There have been few studies on protein glycosylation in the uterine stroma or decidua during the pre- and peri-implantation periods. In mice, glycosaminoglycan was elevated in decidualized uterine horns compared to undecidualized horns (Carson *et al.* 1987). In rats, *N*-linked glycans were found during early decidualization. As decidualization progresses, increased α-2,3-linked sialic acid residues and decreased α-2,6-linked sialic acid residues were observed, suggesting biosynthesis or modulation of existing glycoproteins during decidualization (Jones *et al.* 1993). Pull-down experiments in hESCs found 320 proteins to have different levels of *O*-fucosylation after decidualization (Yang *et al.* 2023*b*). Focusing on *O*-fucosylation of bone morphogenic protein 1 (BMP1), the authors found higher levels of *O*-fucosylated BMP1 in the secretory phase of human endometrium. They also showed that in hESCs, increasing *O*-fucosylated BMP1 enhanced decidualization.

There is also some evidence showing that hyperglycemia can lead to glycation in the decidua. Glycation is a nonenzymatic process where glucose binds to proteins. Some protein glycation is normal, but in the presence of elevated glucose concentrations, advanced glycation end products (AGEs) form (Singh *et al.* 2001). AGEs inhibit normal protein function and interact with receptors, most notably, the receptor for AGEs (RAGE, gene symbol *AGER*) to promote inflammation. In obese patients, immunohistochemistry indicated that AGE levels were increased in the decidua, and RAGE was highly expressed in the uterine epithelium (Antoniotti *et al.* 2018). Accumulation of AGEs could contribute to the impaired decidualization associated with obesity (Rhee *et al.* 2016).

### Glycogen metabolism in the uterine decidua

Given the high levels of glucose catabolism via the PPP glycolysis in the decidua, it might be surprising that decidualization also increases the storage of glucose (Fig. 3). Several studies have found increased glycogen levels in the decidua of both humans and rodents. Glycogen content was increased in the rat uterine horn with developed decidua compared to undecidualized uterine horn (Cecil *et al.* 1962). Similarly, endometrial glycogen content in humans and primates peaks during the secretory phase (Demers *et al.* 1973, Maeyama *et al.* 1977, Mimori *et al.* 1981). While these studies did not localize glycogen, the glycogen was presumably in the decidua. In mice, glycogen content measured with diastase-labile PAS staining was significantly higher in the decidualized stroma at the implantation site compared to undecidualized stroma at the inter-implantation site on day 6. Artificial decidualization also increased the glycogen content compared to undecidualized uterine horn (Chen *et al.* 2022).

Abnormal glycogen metabolism in the decidua has been linked to pregnancy loss. Infertile patients who failed to achieve pregnancy after one year or more for no known reason have lower endometrial glycogen concentrations during the secretory phase (Maeyama *et al.* 1977). A metabolomics study found lower levels of UDP glucose (the substrate for glycogen synthase) in the decidua of women who had experienced recurrent pregnancy loss, which is defined as losing two or more clinically recognized pregnancies before 20 weeks of gestation (Wang *et al.* 2021). A recent study performed single-cell RNA-seq on people with late-onset preeclampsia and normal pregnancy revealed that glycogen synthesis is upregulated in decidualized stromal cells in preeclampsia patients, suggesting that preeclampsia is associated with dysregulation of the decidualized stromal cells (Yang *et al.* 2023*a*).

Decidualization increases both glycogen-synthesizing and -catabolizing enzymes. In humans, the activity of both glycogen synthase and glycogen phosphorylase in the stroma peaked during the secretory phase (Souda *et al.* 1985). Immunohistochemistry indicated that the expression of glycogen synthase was higher in the decidua of pregnant mice and in artificially decidualized uterine horns (Chen *et al.* 2022). Glycogen phosphorylase expression was also increased after decidualization, though to a smaller extent (Bo *et al.* 1964, Chen *et al.* 2022). Interestingly, decidualization also increased glucose-6-phosphatase in mice and rats, indicating that glycogen may be catabolized to glucose and secreted (Christie 1966, Chen *et al.* 2022). More work is needed to understand why the decidua is storing large amounts of glycogen, as it is clearly metabolizing high levels of glucose via other pathways.

## Conclusions and future directions

Glucose is typically thought of as a substrate for glycolysis, producing pyruvate that enters the TCA cycle and powers oxidative phosphorylation to produce ATP. However, it is clear that the epithelium and stroma each metabolize glucose differently to meet their own needs. The epithelium secretes glucose into the uterine lumen and converts glucose into other nutrients (lactate and fructose) for secretion into the lumen to support preimplantation development. The epithelium also uses glucose to glycosylate protein, which is important for embryo attachment. The stroma metabolizes glucose via the PPP to provide necessary substrates associated with decidualization for cell proliferation. The decidua then relies heavily on aerobic glycolysis to produce ATP. The epithelium and decidua can store glycogen, likely to regulate glucose availability as pregnancy proceeds.

More research on factors that control uterine glucose metabolism is needed. The changes in glucose metabolism in cyclic and pregnant animals imply regulation by ovarian hormones. Some research has explored how reproductive hormones affect glucose metabolism, but factors associated with maternal recognition of pregnancy are completely unknown. Additionally, it is clear that changes in maternal metabolism, for example, obesity in humans or lactation in dairy cows, reduce fertility. Changes in metabolic hormones (e.g. insulin) or serum glucose concentrations likely affect how the uterus uses glucose, but how altered physiological states affect uterine metabolism is almost entirely unexplored.

## Declaration of interest

The authors declare that there is no conflict of interest that could be perceived as prejudicing the impartiality of this review.

## Funding

This work was funded by start-up funds provided by the University of Illinois and USDA National Institute of Food and Agriculturehttp://dx.doi.org/10.13039/100005825, Hatch project ILLU-538-949 to MD. It was also supported by 1R01HD111706 from NIH to MD.

## Author contribution statement

ZC drafted the original version of the manuscript. ZC and MD edited and reviewed the manuscript. Both authors approved the final version of the manuscript.
